# Reader technique as a source of variability in determining malaria parasite density by microscopy

**DOI:** 10.1186/1475-2875-5-118

**Published:** 2006-12-12

**Authors:** Wendy Prudhomme O'Meara, Mazie Barcus, Chansuda Wongsrichanalai, Sinuon Muth, Jason D Maguire, Robert G Jordan, William R Prescott, F Ellis McKenzie

**Affiliations:** 1 Division of International Epidemiology and Population Studies, Fogarty International Center, National Institutes of Health, 16 Center Dr., Building 16, Bethesda MD 20892, USA; 2 Hydas World Health, Hershey, PA, USA; 3 U.S. Naval Medical Research Unit No.2, Jakarta, Indonesia; 4 National Center for Parasitology, Entomology and Malaria Control (CNM), Ministry of Health, Phnom Penh, Cambodia; 5 U.S. Naval Medical Research Unit No.2, Jakarta, Indonesia; 6 National Institutes of Health, Bethesda, MD, USA

## Abstract

**Background:**

Accurate identification and quantification of malaria parasites are critical for measuring clinical trial outcomes. Positive and negative diagnosis is usually sufficient for the assessment of therapeutic outcome, but vaccine or prophylactic drug trials require measuring density of infection as a primary endpoint. Microscopy is the most established and widely-used technique for quantifying parasite densities in the blood.

**Methods:**

Results obtained by 24–27 expert malaria microscopists, who had independently read 895 slides from 35 donors, were analysed to understand how reader technique contributes to discrepancy in measurements of parasite density over a wide range of densities.

**Results:**

Among these 35 donations, standard deviations ranged from 30% to 250% of the mean parasite density and the percent discrepancy was inversely correlated with the mean parasite density. The number of white blood cells indexed and whether parasites were counted in the thick film or thin film were shown to significantly contribute to discrepancy amongst microscopists.

**Conclusion:**

Errors in microscopy measurements are not widely appreciated or addressed but have serious consequences for efficacy trials, including possibly abandoning promising vaccine candidates.

## Background

Microscopy has been used to detect malaria parasites in the blood of infected patients since Laveran first identified the parasites in 1880 [1]. Microscopic examination of blood is the most affordable, accessible, widely used and reliable technique for diagnosis of malaria infection. Although molecular techniques for quantifying parasites have made significant progress in recent years, microscopy remains the primary technique for quantification of parasites. Microscopy is routinely relied upon as a primary endpoint measurement for epidemiological studies, intervention studies, and clinical trials. Despite the critical importance of microscopy for the study and treatment of malaria, little effort has been made to precisely determine and distinguish sources of error in microscopic diagnosis and quantification of parasitaemia or to evaluate the impact of this error on endpoint measurements.

Like all detection methods, microscopy is an imperfect technique. However, unlike other methods, such as PCR and immunochromatographic assays, it relies heavily upon the judgment and experience of the individual user. This was noted at least as early as 1930, when Knowles and White reported on the 'Training and experience of the observer, the personal factor in the diagnosis of malaria' [2].

To understand how reader technique contributes to discrepancies in reporting parasite species and densities, results from 895 slides made from 35 blood donations were analysed. Some of the slides were parasite-positive and some were guaranteed parasite-negative donations. One slide from each donation was sent to each of 27 expert malaria microscopists for evaluation of parasite presence, species and density. Each participant was asked to report the number of sexual and asexual stages of each species present, but was permitted to choose the manner in which slides were read. There were considerable differences in the densities and species reported for each sample. Analysis of these results yields important insights into the sources of discrepancies between readers reported elsewhere [3-5] and points to possible unevaluated error in reported microscopy results supporting much of the malaria literature.

## Methods

Details of the patient selection, sample collection and preparation, and reference reader selection and participation have been described in detail elsewhere [6] and are summarized below.

### Sample collection and preparation

Donors were chosen from among symptomatic patients self-presenting to regional health clinics or involved in Internal Review Board-approved malaria research protocols in Cambodia and Indonesia and consent specific to this study was obtained from each participant prior to drawing blood. Malaria-negative donations were taken from individuals who are natives of non-endemic areas who had not been exposed to risk of malaria in the past two years. Approximately 3 ml of venipuncture blood was collected in an ethylene-diaminetetraacetic acid (EDTA)-filled tube and multiple slides (up to 150), with both a thick and a thin smear, were prepared from each donation within hours of sample collection. Thick films were made by spreading exactly 6 μl of blood in a circle of 12 mm in diameter. Two microliters of blood were spread using the edge of a clean slide to make a thin film. The thin film was fixed with methanol and the entire slide was stained with Giemsa solution using standard procedures. Negative donations were stained in separate containers which had never been used for positive donations. Only slides from the same donor were stained in the same batch. Slides were coverslipped to preserve them.

### Density and species determination

Slides from 35 donations were sent to 27 reference readers from 13 countries who were considered skilled malaria microscopists by reputation and who accepted the invitation to participate. Each reader received a slide from each of the 35 donations, but each slide was unique (i.e. no reader observed exactly the same slide). Each reader was asked to record the density of asexual and sexual forms of each species observed on the slide. When it was not possible to distinguish between asexual forms in mixed infections, readers often reported the species observed and recorded a total asexual parasite density. No guidelines were given as to how to count the parasites, whether by co-counting white blood cells (WBC) or co-counting red blood cells (RBC), or how many reference RBCs or WBCs should be reported, although the spreadsheet used by each reader to record data included columns for number of WBCs and number of RBCs. The absence of a rigid counting protocol generated a heterogeneity of counting techniques which permitted us to analyse differences between them.

### Analysis

Results which were reported as percent parasitaemia, percent parasitized RBCs or had no information as to how many RBCs or WBCs were indexed were excluded from this analysis (approximately 2.5% of reads). The number of asexual parasites observed was divided by the number of RBCs or WBCs indexed and multiplied by the standard approximation of 8,000 WBCs per microliter or 4.5 × 10^6 ^RBCs per microliter [7]. Parasite density data were analysed by summing the asexual forms across all the species present and creating a total-parasites-per-microliter value. Non-parametric statistical tests were performed using the Analyze-It tool for Microscoft Excel.

## Results

### Variation between readers

**Figure 1:**
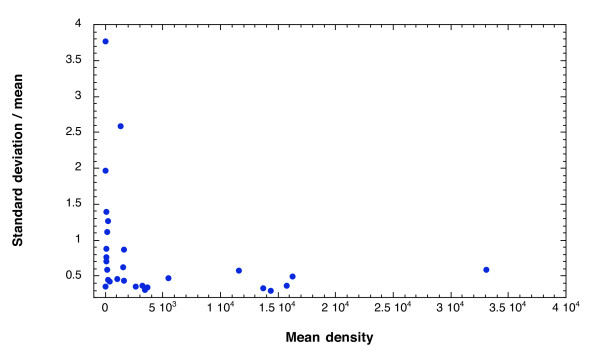
**Standard deviation of parasite density scaled by the mean decreases as the mean density of infection increases**. Standard deviation of reads from 27 malaria microscopists evaluating parasite density from slides made from 30 different donations is scaled by the mean density of each donation and plotted against the mean density.

Differences in results reported by individual readers evaluating different slides from the same donation include differences in individual judgment and technique, and differences which arise due to handling or random distribution of parasites in the blood. Figure 1 shows the standard deviation of density readings scaled by the inter-reader mean density for each donation plotted as a function of the mean density. There is a significant inverse correlation between discrepancy among microscopists and mean density (p = 0.0096, Spearman rank correlation). The median scaled deviation is 0.58.

To further dissect the source of reader discrepancy at very low density, one microscopist examined the entire thick film of two slides from the same donation. He identified 57 parasites on one slide and 49 parasites on the other, giving densities of 10 and 8 parasites/μl, respectively. The original results reported for these same two slides by two other independent readers were 80 parasites/μl and 0 parasites/μl, indicating that random chance in the selection of fields to examine may play a considerable role in reader discrepancy, particularly at low densities.

### Counting technique

Readers were permitted to use either the thick film or the thin film and to count either parasites per WBC or parasites per RBC. There were no guidelines given as to how many WBCs or RBCs should be indexed when measuring the parasite density. Some readers indexed as many as 8,600 WBCs and 150,000 RBCs or as few as 3 WBCs and 400 RBCs (median WBCs and RBCs counted were 203 and 2,000, respectively). 10.4% of all positive results were reported as parasites per RBC.

### Thick film (WBC) method

**Figure 2:**
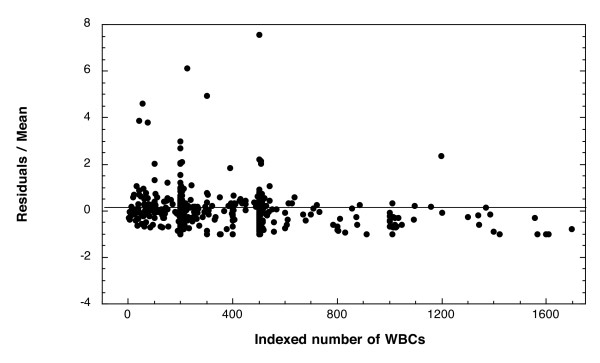
**Deviation from the mean parasite density decreases as the number of WBCs indexed increases**. The residual of each measurement (read of sample Y from microscopist X-mean of all microscopists for sample Y) was scaled by the sample mean and plotted as a function of the number of reference WBCs counted for that read. Reads which were made by indexing RBCs were excluded from this plot, but were included in the sample mean.

In order to understand how the differences in the numbers of reference blood cells counted contributed to overall discrepancy among readers, the first analysis used only counts made from the thick film (WBCs) and excluded counts made from the thin film (RBCs). The difference between the individual reads and the group mean for a donation was scaled by the group mean (residual of reader X for sample Y divided by the mean density of sample Y). This scaled residual was plotted versus the mean density for every measurement of every donation (Figure 2). For all donations, the deviation of the scaled residuals from the mean decreased as the number of reference WBCs increased. There is a significant negative correlation between the magnitude of the residuals and the number of WBCs counted for each measurement (p = 0.0003, Spearman rank correlation).

### Thick film (WBC) method vs. thin film (RBC) method

**Figure 3:**
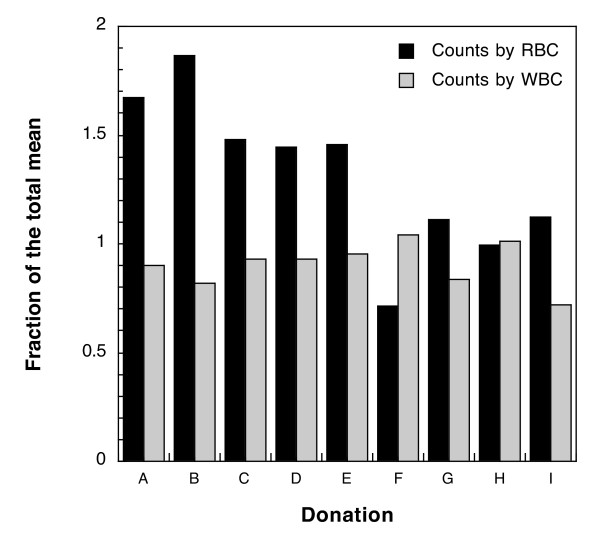
**Parasite density measurements made by counting parasites per RBC in the thin film are consistently higher than measurements made using WBCs in the thick film**. The fold-difference of the mean from RBC-based densities and WBC-based densities compared to the total mean.

Next, the parasite densities reported based on readings from the thick film were compared to those from the thin film. RBCs are lysed during staining of the thick film; therefore, parasite densities reported against a reference number of RBCs could only have been counted in the thin film. The inverse was also assumed to be true, *i.e*. that parasite densities reported as parasites per WBC were always counted in the thick film. There were nine donations for which reads (at least two) were reported from both the thick and thin film. These donations had a higher mean density than donations for which all microscopists counted the thick film (70,613/μl versus 1,569/μl), indicating that microscopists were more likely to choose to count parasites on the thin film as parasite density increased. There were no donations for which all the microscopists counted parasites in the thin film. The mean density among measurements taken from the thick film were compared to those taken from the thin film. Figure 3 shows the ratio of the mean density reported from WBCs to the total mean density and the ratio of the mean density from RBCs to the total mean density for each donation which had more than one instance of each method. The measurements from the thin film averaged about 30% higher than the total mean whereas densities measured from the thick film averaged 10% lower than the total mean. The difference between the thick- and thin-film counts was significant as determined by the Mann-Whitney U test (p = 0.0011). It is possible that this difference could arise from the multiplicand used to convert from RBC or WBC to volume. If the approximation of 4,500,000 RBCs per microliter represents a large overestimate compared to 8,000 WBCs per microliter, this could account for the systematically higher means from RBC counts. There was no consistent trend for gametocyte counts (data not shown). The density of gametocytes is generally much lower than that of asexual forms, and, therefore, the volume of blood examined greatly affects the number observed. The volume of blood per field of a thin film is about 20–30 times less than a thick film, leading to a greatly decreased probability of observing a gametocyte in any given field. In these nine donations, gametocytes were more often observed in the thick film. When gametocytes were identified by readers examining the thin film, the reported density of gametocytes was higher in the thin film than in the thick film.

### White blood cell distribution

**Figure 4:**
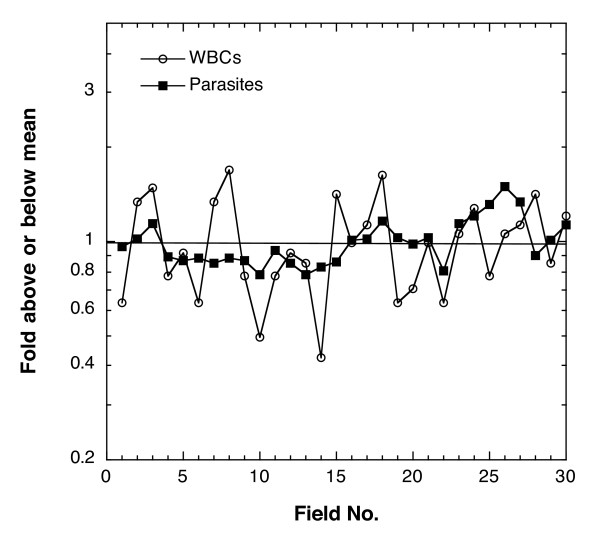
**WBC distribution in the thick film is less uniform than parasite distribution**. The parasites per field and WBCs per field for 30 fields of a thick film are plotted as fold above or below the mean parasites or WBCs per field for all 30 fields.

As demonstrated above, the precision or agreement of the density measurements is strongly dependent on the number of WBCs counted. To determine how uniform the distribution of WBCs is from field to field in the thick film, one microscopist counted the parasites per field and the WBCs per field over 30 high-powered fields of a single thick film. He found a total of 424 WBCs and 8,897 parasites in 30 fields. Figure 4 shows the number of parasites and WBCs per field. Each per-field value is divided by the mean over all 30 fields to give the proportion of parasites or WBCs above or below the mean in each field. The number of parasites per field is more evenly distributed than the number of WBCs per field. A thick film prepared from 6 μl of blood had approximately 2,800 fields, as evaluated by one reader. Calculating the parasite density by adding the total WBCs and the total parasites counted in 30 fields and using the WBC approximation (8,000 WBC per μl) gives a value of 168,000 parasites/μl ([8,897 p in 30 fields/424 WBCs in 30 fields] × 8,000 WBCs/μl = 168,000 p/μl). Using the conversion factor of 2,800 fields per thick film gives a value of 138,000 parasites per μl ([8,897 p/30 fields] × [2,800 fields/6 μl of blood] = 138,000 p/μl), a difference of 18%.

## Discussion

There is a critical and long-standing need for tools to standardize the training and evaluation of malaria microscopists. Slide sets consisting of donations with three species individually or in mixed infections and with a wide range of densities were produced to serve as such a tool [6]. Because microscopy is the only technique available for enumerating parasites in dried blood films, such slides must be validated by the very technique they are designed to improve. The slides intended for these sets were validated by a panel of reference readers with extensive experience in malaria microscopy. Comparing results among such a large panel of experienced microscopists from all over the world was only possible due to the meticulous banking of large numbers of slides prepared simultaneously from each donation. Despite the collective knowledge and experience of the readers, there was considerable discrepancy among them concerning species [6] and densities. This study analysed the contributions of reader technique and method of enumeration to the variability in the density measurements.

Discrepancy among readers decreased with increasing parasite density. This is in agreement with an analysis of field data from Peru and Thailand [5] and from Uganda [4]. Counts from the same slide made by two different microscopists reported by Earle and Perez in 1932 [8] also show a very similar trend with density. The data in these three studies compared the differences between two readings performed on individual samples. The data analysed in this study are unique in the number of readings taken for each sample (n = 24–27) by independent experts. It is the first study analysing more than three quantitative measurements per sample. The median standard deviation of parasite density for a donation was 58% of the mean and ranged from 30% of the mean to 250% of the mean. The deviation among reads of a single sample is attributable to differences in reader technique and judgment as well as differences between slides made from a single blood sample, a complication that is not present in the studies mentioned above where reads were made from the same slide. It was not possible to estimate from these data differences between slides prepared from the same blood sample which arise due to sample handling and preparation, imperfect lysis of RBCs in the thick film, and random distribution of parasites in the blood, but the preliminary data reported above indicate that the latter may be quite significant. The analysis of field data from Peru and Thailand demonstrated that differences between slides made from the same blood sample and read by the same microscopist are not trivial and were, on average, 8–17%. This is a critical issue which remains to be addressed systematically in a manner which will allow the magnitude of the differences between reader technique, differences between slides, and random error to be quantified and compared.

The observation that the deviation from the mean decreased as the number of WBCs indexed by the reader increased is logical. In general, as the number of measurements in any experimental system increases, the sample mean approaches the true mean or the expectation of the mean. Since the parasite density was evaluated as a function of the number of parasites per WBC, the estimate of the true number of parasites per WBC improves as the number of measurements (i.e. WBCs) increases. The validity of this technique is limited by the implicit assumption that WBCs are evenly distributed in the film. To test this assumption, the number of parasites and the number of WBCs per field were counted in a single thick film. Over 30 fields, the WBCs were much less uniformly distributed than the parasites. This leads to the conclusion that, even if the readers identified every true parasite without error, the variation in the distribution of WBCs from field to field could significantly contribute to discrepancy among readers and errors in estimating true parasite density.

Comparing parasite densities observed in thick films to those observed in thin films confirmed the finding of Dowling and Shute [9] and others [10] who reported that as much as 60% percent of parasites were either obscured in the thick film or lost during staining and lysis of red blood cells. The results presented here show that 0–65% fewer parasites were counted in the thick films when compared to the thin film. In only one instance were more parasites (46%) observed by microscopists reading the thick film. One other study [11] found acceptable agreement between thin and thick film density measurements when parasite density counted against RBCs in the thin film was compared to parasite density counted per high powered field (HPF) in the thick film.

This analysis of differences in reader technique suggests several important considerations when attempting to reliably estimate parasite density from blood films. To improve precision, or agreement among readers, a uniform counting protocol should be applied. Increasing the number of indexed cells (WBCs or RBCs) also improves agreement between readers. However, to improve both precision and accuracy, the use of WBCs could be eliminated in favour of a grid counting or HPF counting method which allows the parasites per volume to be counted directly and avoids the problem of unevenly distributed WBCs and an inaccurate conversion factor [11,12]. Using the standard conversion factor of 8,000 WBCs per μl was adequate in this application because reads were only being compared between microscopists examining the same blood sample. For studies where patient parasitaemias are being compared to each other or within a group, the standard WBC approximation is grossly inadequate as WBC counts can vary greatly between individuals and decrease as a result of malaria infection [13]. Applying a volume-counting method or using true WBC counts for each patient as has been done in some clinical trials [14-16] would eliminate this source of error.

## Conclusion

Accurate and precise quantification of malaria parasites is a critical endpoint for many types of intervention trials. In a clinical setting, errors could endanger a patient. Recent studies and unpublished personal experience have shown that false positive and negative rates using microscopy in the field can be as high as 30% [3] and discrepancies in density measurements can be as much as 2–3 fold [5]. This study identified similar discrepancies in ideal laboratory conditions among expert microscopists and demonstrated that at least a portion of the discrepancy is due to differences in reader technique and the use of WBCs as a counting index. It also highlights the importance of reporting slide reading protocols in published studies and the potential danger of comparing density data between studies. More experiments and analyses need to be done to determine how much this problem could be ameliorated by increased training, standardization of techniques and counting protocols, and improved awareness of the errors inherent in microscopic detection and quantification of malaria parasites.

## Conflict of interest

The author(s) declare that they have no competing interests.

## Authors' contributions

WPO contributed to the study design and data analysis and prepared the first draft of the manuscript. MB contributed to the design and execution of the study, data analysis, and writing of the manuscript. CW contributed to the design and execution of the study as well as the analysis and intellectual content of the manuscript. SM contributed to the design and execution of the study. JM contributed to the intellectual content of the manuscript. RJ contributed to the study design and analysis. WRP contributed to the study design, data analysis, and drafting of the manuscript. FEM contributed to the study design, data analysis, and drafting of the manuscript.

## Disclaimer

The views and opinions are those of the authors and do not purport to represent those of the U.S. Navy or Department of Defense.
